# A101 VIRTUAL MINDFULNESS-BASED STRESS REDUCTION FOR ADULTS WITH INFLAMMATORY BOWEL DISEASE: FEASIBILITY TRIAL PRELIMINARY RESULTS

**DOI:** 10.1093/jcag/gwac036.101

**Published:** 2023-03-07

**Authors:** K Chappell, K J Goodman, J -M Le Melledo, D Meakins, M Marsh-Joyal, K I Kroeker

**Affiliations:** University of Alberta, Edmonton, Canada

## Abstract

**Background:**

Patients with Inflammatory Bowel Disease (IBD) often suffer from high levels of anxiety and depression. Despite high rates of mental health comorbidity, a low proportion of patients receive psychiatric referrals and treatment. In Canada, provincial health care plans cover psychiatric services, making them affordable for patients and making referral efficient for gastroenterologists. Psychiatrist-led virtually-delivered Mindfulness-Based Stress Reduction (MBSR) has been associated with reducing feelings of stress, anxiety, and depression in several high-quality randomized control trials. It also reduces cost and travel requirements for patients, both which have been identified as barriers to accessing mental health treatment.

**Purpose:**

To assess the feasibility of online-delivered MBSR for IBD patients, with feasibility outcomes defined as recruitment success, and attendance, adherence, and attrition of participating patients.

**Method:**

Eligible participants were adult IBD patients aged 18-65 attending gastroenterology clinics in Edmonton, Alberta who self-identified as being anxious or depressed and/or were referred by their gastroenterologist. A research coordinator contacted eligible patients who expressed interest in participating after completing an assessment of symptoms and a semi-structured interview with a psychiatrist. The MBSR protocol was an 8-week group-based intervention aimed at giving participants tools to cope with stress effectively. Participants attended 8 weekly sessions lasting 2.5 hours/week and a one-time weekend session lasting 5 hours. They were also asked to practice every night for 45-60 minutes. Completion of the program required attendance of at least 6 of 8 weekly sessions and the weekend session. Two groups, led by the same team of qualified psychiatrists, started MBSR, with sessions occurring in the evening via Zoom.

**Result(s):**

Of the 64 patients referred to the study, 16 (25%) agreed to participate. Reasons for declining to participate are shown in Table 1, with 80% indicating they were too busy. Of the 16 patients enrolled, the median age was 36 (range: 18-55), 10 identified as female (62.5%) and 8 had Crohn’s Disease (50%). Attendance, adherence, and attrition data from the first group of 7 participants were recorded. Only 3 (42.8%) successfully completed the program. The participants that completed the program had an attendance rate of 100% and practiced 6 nights a week for an average of 25 minutes a night. A second group with 9 participants is currently ongoing.

**Image:**

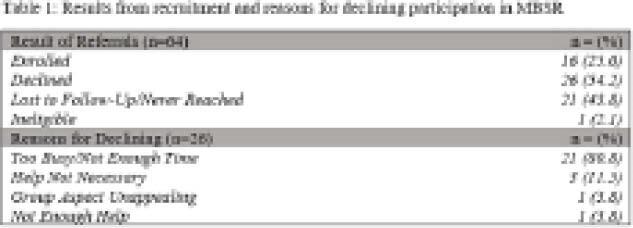

**Conclusion(s):**

Although interest in a cost-free, virtual stress management resource was relatively high, willingness to enroll in MBSR specifically, was low, largely due to the time commitment. Follow-up interviews with those who enrolled and did not enroll in the intervention are underway to highlight the benefits and barriers to MBSR.

**Disclosure of Interest:**

None Declared

